# Design, Optimization, and Application of a 3D-Printed Polymer Sample Introduction System for the ICP-MS Analysis of Nanoparticles and Cells

**DOI:** 10.3390/nano13233018

**Published:** 2023-11-25

**Authors:** Gyula Kajner, Ádám Bélteki, Martin Cseh, Zsolt Geretovszky, Tibor Ajtai, Lilla Barna, Mária A. Deli, Bernadett Pap, Gergely Maróti, Gábor Galbács

**Affiliations:** 1 Department of Molecular and Analytical Chemistry, University of Szeged, Dóm Square 7-8, H-6720 Szeged, Hungary; 2 Center of Excellence for Interdisciplinary Research, Development and Innovation, 3D Center University of Szeged, Tisza Lajos Boulevard 107, H-6725 Szeged, Hungary; 3 Department of Optics and Quantum Electronics, University of Szeged, Dóm Square 9, H-6720 Szeged, Hungary; 4 ELKH-SZTE Research Group for Photoacoustic Monitoring of Environmental Processes, Dóm Square 9, H-6720 Szeged, Hungary; 5 HUN-REN Biological Research Centre, Institute of Biophysics, Temesvári Boulevard 62, H-6726 Szeged, Hungary; 6 HUN-REN Biological Research Centre, Institute of Plant Biology, Biological Research Center, Temesvári Boulevard 62, H-6726 Szeged, Hungary

**Keywords:** nanoparticle analysis, single cell analysis, 3D-printing, sample introduction, single particle ICP-MS

## Abstract

Commonly used sample introduction systems for inductively coupled plasma mass spectrometry (ICP-MS) are generally not well-suited for single particle ICP-MS (spICP-MS) applications due to their high sample requirements and low efficiency. In this study, the first completely 3D-printed, polymer SIS was developed to facilitate spICP-MS analysis. The system is based on a microconcentric pneumatic nebulizer and a single-pass spray chamber with an additional sheath gas flow to further facilitate the transport of larger droplets or particles. The geometry of the system was optimized using numerical simulations. Its aerosol characteristics and operational conditions were studied via optical particle counting and a course of spICP-MS measurements, involving nanodispersions and cell suspensions. In a comparison of the performance of the new and the standard (quartz microconcentric nebulizer plus a double-pass spray chamber) systems, it was found that the new sample introduction system has four times higher particle detection efficiency, significantly better signal-to-noise ratio, provides ca. 20% lower size detection limit, and allows an extension of the upper limit of transportable particle diameters to about 25 µm.

## 1. Introduction

Inductively coupled plasma mass spectrometry (ICP-MS) is a widely used, high-performance atomic spectroscopy method with great versatility in ultratrace element and stabile isotope analysis [1]. Single particle ICP-MS (spICP-MS) is an ICP-MS-based technique that was initially described by Degueldre et al. in 2003 [2]. As of today, it has become an increasingly popular particle characterization technique that is able to detect individual particles based on their elemental or isotopic content and provide valuable information about the number concentration and size distribution of suspended nanoparticles (NPs) [2,3,4]. In addition to the basic characterization of NPs, the spICP-MS technique has been shown to be capable of also providing information about the structure, shape (aspect ratio), porosity, or density of NPS [5,6,7], but compositional information about alloy NPs and the load concentration of nanocomposite catalysts was also successfully determined by this technique [6,8]. However, years before the popularization of the single particle ICP-MS technique for nanoparticle characterization, in 1994, Nomizu et al. had already demonstrated the determination of the total calcium content in individual mammalian cells using the same principle [9]. This approach, later often referred to as single cell ICP-MS (scICP-MS), has proved to be a potent tool for researchers exploring cell-to-cell variation of elemental concentration levels or certain metabolic uptake routes in a wide variety of cell cultures [10,11,12]. Therefore, there has been a growing interest in the past two decades in the scICP-MS technique across various research fields of biology and bioinorganic chemistry such as oncology, microbiology, nanotoxicology, pharmacology, etc. [10,11,13].

All in all, it is fair to say single particle ICP-MS is a versatile and powerful technique to characterize a wide variety of nano- and microparticle suspensions. However, the standard sample introduction system—which has also been called the Achilles’ Heel of atomic spectroscopy—is not ideal for single particle analysis [14,15,16,17]. These systems, which conventionally consist of a pneumatic nebulizer coupled with a double-pass or cyclonic type spray chamber, usually operate at liquid flow rates of 0.1–1 mL·min^−1^ and hardly produce transport efficiencies exceeding 5% [14,18,19]. For conventional, solution-based ICP-MS measurements, this is an intended feature that guarantees stable aerosol output with a well-defined and fine droplet size, facilitating analysis with high sensitivity and high precision [20].

However, utilizing these standard sample introduction systems (SISs) in spICP-MS measurements comes with a rather low particle detection rate that can substantially increase sample consumption and the analysis time. To overcome these difficulties, several high-efficiency sample introduction systems have been developed in the past [21,22]. These systems mostly consist of a nebulizer with modified (decreased) nozzle dimensions to be suited for operation at low sample flow rates (10–100 μL·min^−1^) (e.g., high-efficiency nebulizer (HEN), microconcentric nebulizer (MCN) or micromist nebulizer (MMN)) coupled with a single pass or low volume, cyclonic spray chamber [20,21,23,24]. Although these systems were originally designed to introduce solution samples, lately it has been shown that utilizing such high-efficiency sample introduction systems offers several advantages in spICP-MS analysis: the ability to handle microsamples, improved LODs, decreased background noise, and required sample volume [18,22,23]. This field has drawn considerable attention in the past years, and as a result, several in-depth papers about the application and characterization of such systems have been published [17,18,22].

Despite the obvious benefits that comes with additive manufacturing techniques, attempts at 3D printing parts of sample introduction systems (nebulizers [25,26] and spray chambers [27,28]) have appeared in only a handful of studies so far. The main reason for this is that only state-of-the-art 3D printers have the resolution required to print the microstructures of a nebulizer nozzle with satisfactory precision. For this reason, cross-flow nebulizers are preferred in such studies, which are less challenging from the perspective of manufacturing [25,26]. So far, the only 3D-printed concentric nebulizer was reported by Geertsen et al. in 2020, which had its inner (liquid) capillary prepared separately by other manufacturing techniques and was fixed in place after printing [29].

However, by utilizing high-resolution MultiJet Printing (material jetting) technology [30,31], recently we have shown that fully 3D-printed microconcentric nebulizers can be practically fabricated and demonstrated their successful use for solution ICP-MS analysis [32,33]. The goal of the present study was to use our formerly developed 3D-printed polymer microconcentric nebulizer and complement it with a 3D-printed polymer spray chamber with the purpose of optimizing and assessing the performance of this integrated construction for spICP-MS analysis of nano- and microparticle dispersions.

## 2. Materials and Methods

### 2.1. Instrumentation

An Agilent 7700X (Agilent, Santa Clara, CA, USA) inductively coupled plasma mass spectrometer (ICP-MS) was used in all experiments with 15 L·min^−1^ plasma gas flow rate, 1550 W R.F. forward power, and 10 mm plasma sampling depth. The standard SIS consisted of a MicroMist quartz pneumatic nebulizer (Agilent, Santa Clara, CA, USA) coupled with a Peltier-cooled Scott spray chamber (Port Melbourne, VC, Australia) and was operated with 1.05 L·min^−1^ carrier gas flow and 750 μL·min^−1^ sample uptake rate. During spICP-MS measurements, the data acquisition software was used in time-resolved analysis (TRA) mode, and the integration time (dwell time) was set to 6 ms in the case of nanodispersions and to 12 ms in the case of cell suspensions. Whereas for the measurement of solution samples, the data acquisition software was switched to analog mode, with the integration time set at 500 or 1000 ms. The acquisition time was 60 s. In all experiments, three repetitive measurements were carried out for the purpose of repeatability calculation (RSD%). LMT-55 Tygon pump tubing (Saint-Gobain, Courbevoie, France) with 0.13 mm inner diameter was used for the aspiration of liquid samples when measurements with microflow sample introduction were performed.

Algal cells were observed under an Olympus Fluoview FV-300 type confocal laser scanning microscope (Olympus Optical Co., Ltd., Tokyo, Japan), whereas endothelial cells were observed and counted using a Motic AE2000 type (Kowloon City, Hong Kong) scientific microscope.

Before dilution and directly before aspiration into the ICP-MS, nanoparticle and algal cell dispersions were sonicated in an ultrasonic bath for 5 min (Bransonic 300, Danbury, CT, USA) to eliminate particle aggregation. For endothelial cells, a gentler homogenization procedure involving intensive mixing had to be applied. The conditions and intactness of the cells within the suspensions at different stages of the sample preparation and sample introduction process were inspected, using an Optika Ti600-FL type scientific microscope (Optika S.r.l., Ponteranica, Italy). To reduce the solute content of the media of the algal cell suspensions, each was centrifuged for 5 min at 10,000 rpm in Eppendorf tubes and had its supernatant replaced by water. This process was repeated five times consecutively. Centrifugation was carried out on a Costar Model 10 table-top centrifuge (Corning Costar Co., Corning, MA, USA).

The aerosol characteristics of the SISs were assessed using a model 1.109 optical particle counter (OPC) (Grimm Aerosol Technik, Ainring, Germany).

### 2.2. Samples and Materials

Trace-quality de-ionized water from a MilliPore Elix 10 device equipped with a Synergy polishing unit (Merck, Darmstadt, Germany) was used for the preparation of all solutions and diluted suspensions. High-purity (99.996%) technical argon gas was used in the experiments, obtained from Messer (Messer Hungarogáz, Hungary).

Cobalt solutions were prepared from 1000 mg·L^−1^ CertiPUR monoelemental standard (Merck, Germany). In the spICP-MS experiments related to nanoparticles, dispersions of spherical gold (average diameters: 20.8, 28.0, 39.0, 58.0, 83.0, and 100.9 nm; obtained from nanoComposix Inc., San Diego, CA, USA) and silver (average diameter: 59.0 nm; obtained from Ted Pella, Redding, CA, USA) were used.

**Figure 1:**
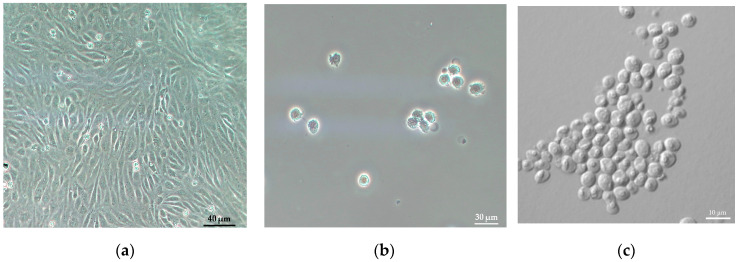
Illustrative microscopy images of the cells studied. Phase contrast microscopy pictures of human endothelial cells as confluent monolayers (**a**) and as dispersed cells after paraformaldehyde fixation (**b**). Confocal laser scanning microscope picture of *Chlorella* sp. Algal cells (**c**).

In the experiments involving algae cells, one strain of microalgae was used to produce suspensions, namely *Chlorella* sp. (MACC-360), which was obtained from the Mosonmagyaróvár Algae Culture Collection (MACC) (Figure 1c). All microalgae were cultivated at 25 °C under a light intensity of 50 µmol·m^−2^·s^−1^ with continuous shaking at 180 rpm in a regime of 16:8 light–dark periods. All cell culture was grown in TAP medium, which consisted of 2.42 g·L^−1^ of Tris base, 0.374 g·L^−1^ of NH_4_Cl, 0.204 g·L^−1^ of MgSO_4_·7H_2_O, 0.066 g·L^−1^ of CaCl_2_·2H_2_O, 0.287 g·L^−1^ of K_2_HPO_4_, 0.142 g·L^−1^ of KH_2_PO_4_, 0.049 g·L^−1^ of Na_2_EDTA·2H_2_O, 0.039 g·L^−1^ of ZnSO_4_·7H_2_O, 0.011 g·L^−1^ of H_3_BO_3_, 0.007 g·L^−1^ of MnCl_2_·4H_2_O, 0.008 g·L^−1^ of FeSO_4_·7H_2_O, 0.002 g·L^−1^ of CoCl_2_·6H_2_O, 0.002 g·L^−1^ of CuSO_4_·5H_2_O, 0.001 g·L^−1^ of (NH_4_)_6_Mo_7_O_24_·4H_2_O and 1 mL·L^−1^ of CH_3_COOH and the pH was maintained at 7. To study the effects of Ni^2+^ ions on microalgae cultures, media with additional 50, 100, 150 μM of Ni^2+^ concentrations were made using an analytical grade standard nickel solution.

Human vascular endothelial cells (passage 7) were grown in endothelial cell culture medium (ECM) (Sciencell, Carlsbad, CA, USA), supplemented with 5% fetal bovine serum, 1% endothelial growth supplement (Sciencell, Carlsbad, CA, USA) and 0.5% gentamicin (Merck Life Science Ltd., Budapest, Hungary) as described previously [34]. Cells were seeded into collagen type IV and fibronectin coated (100 µg/mL each; Merck Life Science) Petri dishes (60 mm; Corning Costar Co., Corning, MA, USA). When cells reached confluence, they were washed with ECM medium, dissociated with trypsin-EDTA solution (Merck Life Science), and centrifuged at 400 g for 5 min. The cell pellet was resuspended and fixed in 1% paraformaldehyde solution (Merck Life Science) for 20 min at 4 °C. Cells were centrifuged again at 400 g for 5 min and diluted in phosphate-buffered saline solution (pH 7.4) for further experiments (Figure 1a,b).

### 2.3. Software

All ICP-MS data processing was performed within the Agilent MassHunter (Agilent Technologies, Santa Clara, CA, USA), Origin (OriginLab Corp., Northampton, MA, USA), and MS Office Excel (Microsoft Corp., Redmond, WA, USA) software. Hydrodynamic simulations on flow conditions were performed utilizing the COMSOL Multiphysics software package (COMSOL Inc., Burlington, MA, USA), in which the 3D models of the sample introduction systems were created utilizing the AutoCAD (Autodesk Inc., San Francisco, CA, USA) and Solid Edge (Siemens PLM Software, Plano, TX, USA) engineering design software. During spICP-MS data evaluation, background peaks were fitted with an asymmetrical Poisson function, while the particle peaks were fitted with a lognormal curve. The count value associated to a particle of a given size (mass) was derived by subtracting the mode of the background peak from the mode of the particle peak.

### 2.4. 3D Printing

3D-printing was done using a ProJet MJP 3600 MAX type professional 3D-printer equipped with a cartridge of VisiJet M3 resin (3D Systems, Rock Hill, SC, USA). The printer was used in XHD mode, in which the nominal layer height is 16 µm and the nominal x and y resolution is 750 dpi. To remove the remainders of the support resin (VisiJet S300) from the surface, all prints were put in a heated laboratory oven stabilized at 75 °C. After most of the wax melted and drained, the parts were placed into a sonicated bath of EZ Rinse liquid (3D Systems, Rock Hill, SC, USA) tempered to 65 °C for 10 min, which was followed by sonication at 65 °C in a detergent solution for another 10 min. Finally, the prints were rinsed with trace analytical purity lab water and dried. In the case of the nebulizer, the heated oven was not used. Instead, pressurized argon gas flow was introduced into the nebulizer during the EZ Rinse liquid bath procedure to prevent the molten support resin or the oil from being sucked into the capillaries.

## 3. Results and Discussions

### 3.1. Development and Fabrication of the SIS

When selecting the most suitable spray chamber design, several considerations were considered. The design had to be one that (*i*) can be operated with flows which are not changing the plasma conditions drastically and can be supplied by the ICP-MS instrument, (*ii*) is directly connectable to the transport tube of the instrument, (*iii*) is dimensionally compatible with our concentric, 3D-printed nebulizer [33], (*iv*) facilitates higher transport efficiencies and promotes the transmission of droplets with diameters exceeding 10 μm, (*v*) has a low dead volume in order to decrease memory effects, and (*vi*) does not contain details which are too challenging for 3D printing.

**Figure 2:**
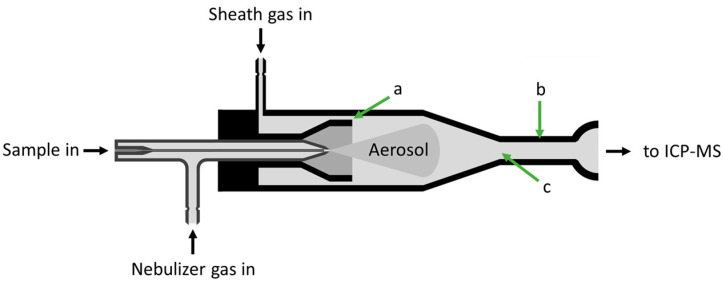
Schematic representation of the sample introduction system. (a) the radial distance between two walls of the spray chamber (sheath gas slit), (b) the length and (c) the width of the cylindrical exit tube.

Based on these considerations, a single-pass type spray chamber was selected due to its straightforward design which was highly compatible with both the ICP-MS instrument and our nebulizer. Furthermore, the design can be complemented with a sheath gas flow which apart from increasing the transport efficiency of the system, also promotes the transmission of droplets with diameters up to tens of microns. The conceptual drawing of this system is shown in Figure 2.

Several hydrodynamic numerical simulations were carried out in COMSOL with the aim of assessing the pressure and flow conditions inside the spray chamber in each of its iterations. These simulations were also used to assess possible droplet trajectories and the approximate transport efficiency for droplets with a wide range of diameters using different sheath gas flow rates.

Variables of the geometrical iterations were the width of the sheath gas slit (from 150 to 750 μm) as well as the length and width of the exit tube (varied from 15 to 30 mm and 3 to 5.5 mm, respectively). All simulations were done on the gas space of the spray chamber (nebulizer was not included except for its orifice) using the combination of the *creeping flow* and *droplet sprays in fluid flow* modules.

First, the performance of different geometrical iterations was assessed, using the following parameters: nebulizer gas flow rate 1.05 L·min^−1^, sheath gas flow rate 0.25 L·min^−1^, outlet pressure condition: 0 Pa (over)pressure. The pressure and velocity conditions, and the transmission characteristics of different iterations showed it was preferable to have as small a sheath gas slit width as practical, as this led to higher gas velocities, which led to increased drag forces that prevent the droplet deposition onto the chamber’s walls. The geometry of the exit tube, in the studied range, was found to be not important from the point of view of the transmission of droplets smaller than 14 μm. However, the simulations also showed for droplet sizes higher than 17 μm, a short and wide exit tube was preferable, as above this size the drag forces progressively fell short in comparison to inertial and gravitational forces. For these reasons, we fixed the sheath gas slit width at 150 μm, and the length and width of the exit tube at 15 mm and 5.5 mm, respectively, in the final geometry of the spray chamber.

**Figure 3:**
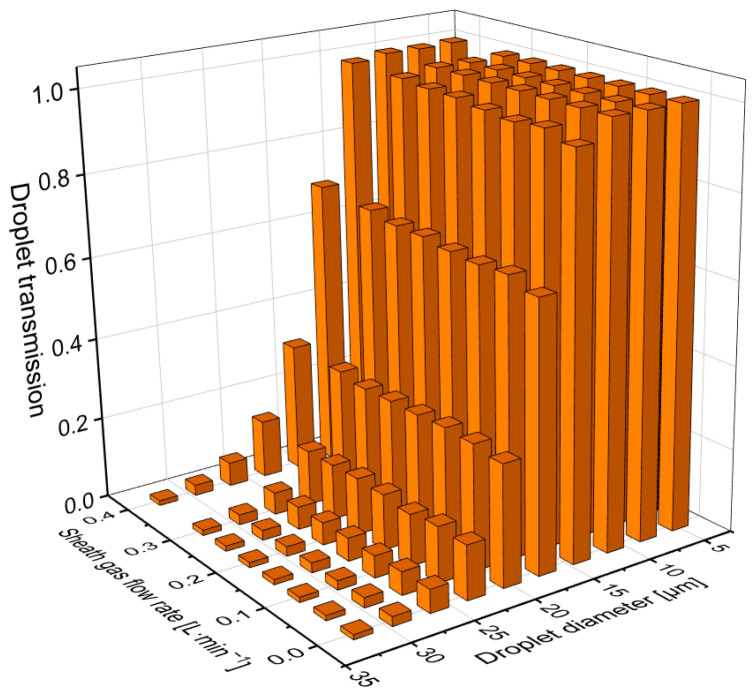
Simulated droplet transmission efficiency values of the sample introduction system for droplet sizes between 5 and 32 μm at different sheath gas flow rates ranging from 0 to 0.4 L·min^−1^.

Next, we investigated how the sheath gas flow rate impacted the transmission of different size droplets. Several time-dependent studies were carried out, each simulating the trajectories of 6000 uniform sized droplets within a 1500 ms time window using 3 ms time steps. The sheath gas flow and the droplet size varied from 0 to 0.4 L·min^−1^ and from 5 to 32 μm, respectively. The latter range was chosen as this started above the typical cut-off droplet size for conventional spray chambers used in ICP-MS practice and basically covered the range of interest for single cell ICP-MS studies. The droplet transmission was defined as the fraction of the droplets in the corresponding simulation that has reached the outlet orifice of the spray chamber. All other parameters were kept constant and the same as in the previous simulations. Figure 3 summarizes the obtained data and indicates the sheath gas does help improve the overall transmission of the sample introduction system. However, its impact is only discernible at droplet sizes between 14 and 26 μm but is most prominent at droplets not bigger than 20 μm in diameter, where 2 to 6% raise in transmission can be observed. Although the effect of the sheath gas was proportional to the flow rate throughout the examined interval, in real experiments keeping the flow rate relatively low comes with obvious benefits too. For instance, it is easier to keep the flow conditions in the laminar regime, and the ion source (plasma) of the instrument is not that severely affected by the cooling effect of the additional gas input.

Thus, although the simulated dataset suggests that using as high a sheath gas flow rate as possible is preferable from the point of view of maximizing the droplet transmission, it is better if in practice it is optimized for each analytical task at hand, e.g., in the case of true solution, nanoparticle, or microparticle (cell) analysis.

As our previous study has shown [33], our nebulizer generates droplets with diameters as high as 30 μm. This, combined with the results of the simulations, which state that droplets with sizes higher than 15 μm have a significant chance of deposition even in ideally laminar conditions, means that during longer measurements one should expect a non-negligible amount of liquid buildup inside the spray chamber. Thus, although most single pass spray chambers lacked this [21,24], a drain channel has been added to our design to handle this problem.

**Figure 4:**
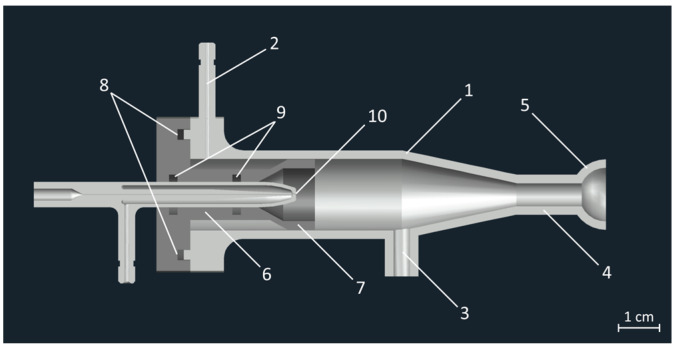
The longitudinal cross-section of the 3D model of the complete sample introduction system. Annotations shows the walls of the spray chamber (1), sheath gas inlet (2), drain channel (3), outlet channel (4), special spherical joint compatible with the transfer tube of the ICP-MS instrument (5), nebulizer’s socket (6), sheath cup (7), O-ring fitting that seals the spray chamber’s two parts (8), and grooves for O-rings (9), which keep the microconcentric nebulizer (10) in position.

**Figure 5:**
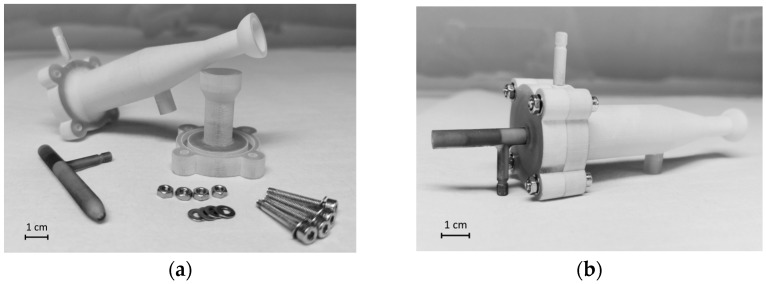
The new sample introduction system in disassembled (**a**) and assembled (**b**) state.

It is also worth mentioning MultiJet 3D-printing technology uses a waxy support resin with which the instrument supports any overhanging part of the model during the printing process. Although the removal of the said support resin was routinely done, the surfaces which were in direct contact with it, were left with altered wetting properties, thus making the surface more susceptible to droplet deposition and water buildup, leading to enhanced memory effects. For this reason, the spray chamber was produced in two parts to allow the printing to be done in an orientation which avoided the contact between the inner walls and any support resin. Figure 4 shows the longitudinal cross-section of the 3D model of the complete sample introduction system. One part contains the walls of the chamber (1), the inlet for the sheath gas (2), the drain channel (3), and the outlet channel (4) with a joint (5) compatible with the transfer tube of the ICP-MS instrument, while part B contains the socket for the nebulizer (6), and the cup (7) that—when assembled—creates the circular slit for the sheath gas flow. To ensure an even sheath slit size along the inner walls and an airtight fit between the two parts of the spray chamber, four M3 bolts and a dedicated fitting (8) with an O-ring were used, while the microconcentric nebulizer (10) was held in place with two 5.5 mm O-rings (9) to ensure a reproducible, centralized nozzle position. Figure 5 shows the sample introduction system in the assembled and disassembled states.

### 3.2. Performance Assessment

Several ICP-MS experiments with the 3D-printed sample introduction system were conducted using solutions, nanodispersions, and cell suspensions with the aim of optimizing the operational parameters of the new sample introduction system. The parameters optimized were the (liquid) sample uptake rate, and the nebulizer gas and sheath gas flow rates. The two quantities used to describe the system’s efficiency are defined below.

For true solution samples, the quantity used to describe the performance of the sample introduction is referred to as signal efficiency. This was defined as

(1) $$\theta =\frac{I}{c_{s}\;Q_{v}}$$

where *θ* is the signal efficiency, *I* [counts·s^−1^] is the ICP-MS analyte signal intensity when a *c_s_* [pg·µL^−1^] concentration sample is introduced at a liquid rate of *Q_v_* [μL·s^−1^]. Please note this measure is inherently affected not only by the droplet formation and transport that takes place inside the sample introduction system, but also by the ionization efficiency of the plasma. The latter is expected to change as the flow rates associated with the sample introduction system (sample, nebulizer gas, sheath gas) vary. However, since sensitivity for liquid sample analysis is just as important as high efficiency aerosol transmission, we chose to focus on their overall effect.

For particle dispersions/suspensions, the particle detection efficiency (also known as transport efficiency in the spICP-MS literature) was used as the metric to describe how well the sample introduction system was functioning with a given set of the three operational parameters. Essentially, this measure is the detected particle fraction and hence is defined as

(2) $$\eta =\frac{N}{N_{NP}\;t\;Q_{v}}$$

where *η* is the dimensionless particle detection efficiency, *N* is the number of particles detected in *t* [s] time, while *N_NP_* [mL^−1^] is the number concentration of the sample which is introduced to the instrument at a volumetric flowrate of *Q_v_* [mL·s^−1^].

Since instrument parameters, such as sensitivity, can change over time, all described quantities are essentially time-dependent. For this reason, the graphs we show about the optimization process use a scale relative to a value which is obtained at the start of each measurement session, using a certain set of parameters (sample uptake: 14.3 μL·min^−1^, nebulizer gas flow rate: 1.07 L·min^−1^, sheath gas flow: 0.0 L·min^−1^).

#### 3.2.1. Performance with True Solutions

**Figure 6:**
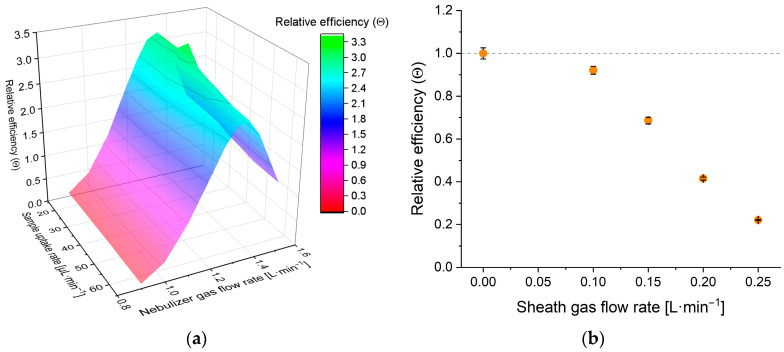
(**a**) Relative signal efficiency (*θ*) values at different sample flow and nebulizer gas flow rates. For graph (**a**), the reference point for the relative scale is the signal efficiency value obtained at 14.3 μL·min^−1^ sample uptake rate and 1.07 L·min^−1^ nebulizer gas flow rate with no sheath gas flow during instrument tuning. The RSD was below 5% for all points, with an average value of 1.7%. (**b**) Relative signal efficiency (*θ*) at different sheath gas flow rates. For graph (**b**), the reference point for the relative scale Is the signal efficiency value obtained at 33.3 μL·min^−1^ sample uptake rate and 1.35 L·min^−1^ nebulizer gas flow rate with no sheath gas flow. Both graphs’ data are from the signal of a 50 ppb Co solution with 1 s integration time. Error bars are based on ten repetitions.

Although our primary goal was to enhance particle detection capabilities, the system is also suitable for the introduction of true solution samples. The effect of the operational parameters on the performance was assessed by using a 50 pbb Co standard solution and an integration time of one second (Figure 6). The signal efficiency values of the system were determined in the parameter matrix of 14.3–64.9 μL·min^−1^ × 0.9–1.5 L·min^−1^ (sample uptake rate × nebulizer gas flow rate), in a total of 7 × 9 points. No sheath gas flow was used in these experiments (Figure 6a).

The experimental results indicate both the sample uptake and the nebulizer gas flow rates have a significant impact, and the latter is far greater. As the nebulizer gas flow increases, the relative efficiency increases and reaches a maximum at 1.35 L·min^−1^ and then starts to decrease. The initial increase in efficiency is most likely a consequence of the increasing gas kinetic energy per unit liquid volume, which leads to finer droplets, thus better transmission. The subsequent decrease on the other hand is most likely due to the combination of at least three effects: the increased cooling effect of higher nebulizer flow rates on the plasma which decreases the ionization efficiency, the slightly shifted optimal sampling depth, and the decreased residence time of the sample.

At the same time, as the sample uptake rate increases, a slow and steady decline in signal efficiency can be observed. This is presumably caused by the cooling effect on the plasma and the decreasing kinetic energy per unit volume of the liquid sample. However, one parameter combination (33.3 μL·min^−1^, 1.35 L·min^−1^) produced a somewhat outstanding (ca. +10% rel.) signal efficiency value (Figure 6a). This is a verified, persistent phenomenon which is most likely caused by the occurrence of some special flow condition resulting from the geometry of the nozzle. Regardless, as this parameter combination consistently produced the highest efficiency, it was used for every following experiment as the optimum.

Effects of the sheath gas flow on the signal efficiency were also examined. As can be seen in Figure 6b, the signal efficiency cannot be increased by utilizing sheath gas. This is presumably a result of the cooling effect on the plasma which is caused by the additional gas flow and the elevated liquid transmission that comes with it.

#### 3.2.2. Performance with Dispersions

Since particle detection substantially differs in nature from regular, solution-based measurements, some parameters expectedly have different effects on the measurement results. Sample uptake and nebulizer gas flow rate, however, have most likely no such effect, since particle sizes below the average droplet diameter essentially do not affect either the formation or the transmission of the aerosol droplets, thus the transmitted volume fraction of the liquid sample and the particle detection efficiency should not differ substantially. Therefore, the previously optimized sample uptake and the nebulizer gas flow rate values were taken over from the previous chapter.

**Figure 7:**
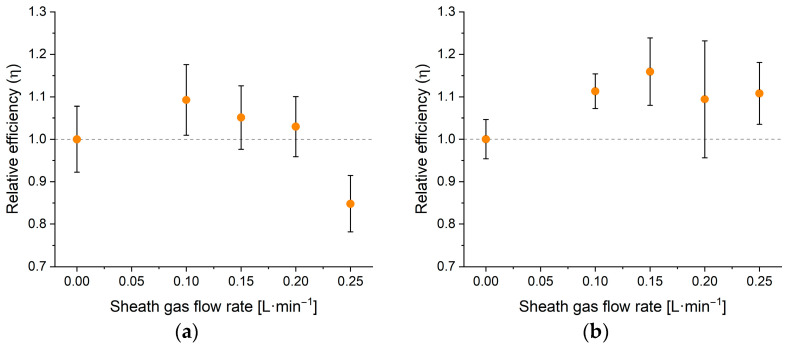
The trends of the relative particle detection efficiency at different sheath gas flow rates when measuring a gold nanoparticle (d_mean_ = 58 nm) dispersion (**a**) and a Chlorella (d_mean_ = 3.6 μm) cell suspension (**b**). The reference point for the relative scale of both graphs is the signal efficiency value obtained by a measurement of the corresponding sample at 33.3 μL·min^−1^ sample uptake rate and 1.35 L·min^−1^ nebulizer gas flow rate with no sheath gas flow. Error bars are based on three repetitions.

On the other hand, sheath gas acts differently in spICP-MS measurements, as the particle detection efficiency (droplet transmission) can be enhanced by utilizing the sheath gas. This is apparent from Figure 7 that shows transport efficiencies of the system (relative to the case when no sheath gas is used) when analyzing a 58 nm Au NP dispersion (a) and a 3.6 μm algal cell dispersion (b). Algal cells were detected at mass 24 exploiting their substantial Mg content. This modest enhancement can be observed because the cooling effect that the sheath gas has on the plasma only affects the signal intensity (sensitivity) of the particles, but not the frequency of detection events, which is described by particle detection efficiency. However, Figure 7 also indicates the sheath gas’ effect on the particle detection efficiency cannot be increased significantly using gas flow rates exceeding 0.1 L·min^−1^. This is also supported by additional experiments conducted with an optical particle counter, allowing us to examine the aerosol output of the sample introduction system at different sheath gas flow rates. Based on these results, 0.1 L·min^−1^ sheath gas flow was found to be optimal for analytical tasks that required the maximization of the detection frequency, as further increasing its value did not enhance the aerosol output. On the other hand, for measurements where maximizing sensitivity is the primary goal, sheathless operation is best.

### 3.3. Comparison with the Standard Sample Introduction System

Several experiments were conducted with the aim of comparing the capabilities and features of the original and the new sample introduction system, mainly focusing on the performance and capabilities regarding single particle detection. In all experiments, the original sample introduction system was operated with its default settings: 750 μL·min^−1^ sample uptake rate, and 1.05 L·min^−1^ nebulizer gas flow rate, while the new system was operated using the parameter set that previously was shown to be optimal for particle detection: 33.3 μL·min^−1^ sample uptake rate, 1.35 L·min^−1^ nebulizer, and 0.1 L·min^−1^ sheath gas flow rate.

#### 3.3.1. Aerosol Characteristics

**Figure 8:**
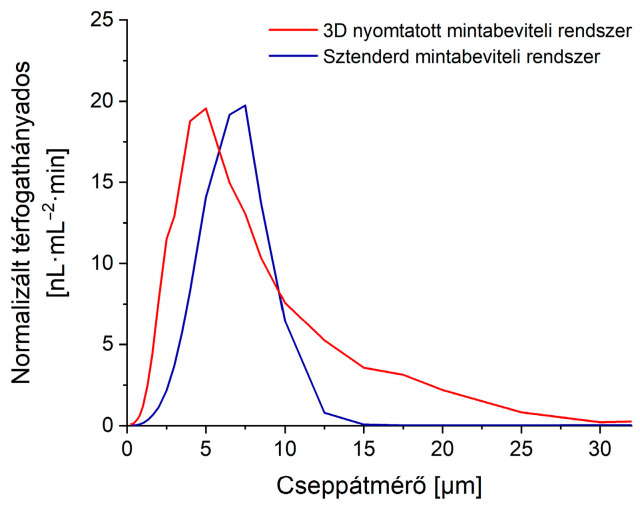
OPC data on the droplet size distribution of the aerosol, weighted with droplet volume, normalized with total liquid volumetric flow rate leaving the exit tube, thus representing the distribution of droplet sizes through which the fluid transport was realized. Operational parameters for the new SIS were 33.3 μL·min^−1^ sample uptake rate, 1.35 L·min^−1^ nebulizer, and 0.1 L·min^−1^ sheath gas flow rate, while for the old system they were set to 750 μL·min^−1^, 1.05 L·min^−1^, respectively, with no sheath gas.

First, the characteristics of the aerosol output of the two systems were examined, using an OPC instrument. For each system, thirty droplet distributions were recorded and averaged. The results are shown in Figure 8.

The graph indicates the new SIS has a considerably lower mean droplet size, meaning a significant fraction of droplets has a higher chance of reaching the plasma, which makes the new system more efficient. Also, while the standard system has its cut-off at around 12.5 μm, the new system allows for the transmission of a wider range of droplets, making the detection of larger particles/cells, up to around 25 μm, possible.

#### 3.3.2. Relative Particle Detection Efficiencies

We also compared the particle detection efficiency of the standard and the new sample introduction system using d = 58 nm gold, and d = 59 nm silver nanoparticle dispersions, along with a d = 3.6 (0.302) μm Chlorella algae dispersion.

For gold and silver nanoparticles, the particle detection efficiency of the standard system was found to be 3.47 (0.025)% and 3.30 (0.087)%, respectively. The new SIS showed a significantly improved efficiency in comparison, namely 12.05 (0.47)%, and 12.35 (0.25)%, respectively. The nearly four-fold improvement in the particle detection efficiency, along with the more than one magnitude lower sample uptake rate, makes the new system better suited for spICP-MS analysis, as well as for the analysis of microsamples.

When measuring the cell suspension, the new system produced only about 1.7 times higher particle detection efficiency than the original one. This result is in accordance with the data in Figure 8, since droplets with sizes below the average size of the cells can not contribute to the detection events, therefore causing a decrease in the particle detection efficiency.

**Figure 9:**
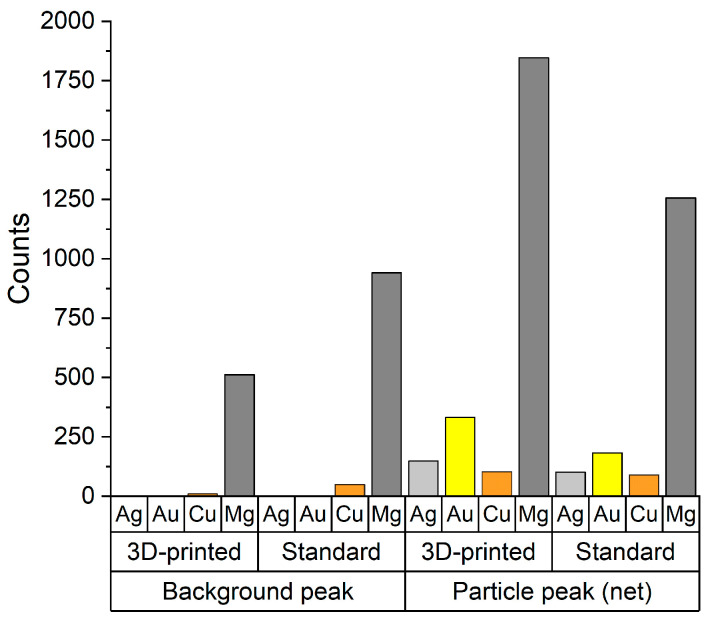
The mean position of particle and background peaks on the TRA signal histogram of various samples, which were analyzed using both the 3D-printed and the original sample introduction system. Ag: peaks of a d_mean_ = 59 nm, silver nanoparticle dispersion, Au: peaks of a d_mean_ = 58 nm, gold nanoparticle dispersion, Cu, Mg: peaks of a d_mean_ = 3.6 μm Chlorella cell suspension, detected at ^63^Cu and ^24^Mg.

**Figure 10:**
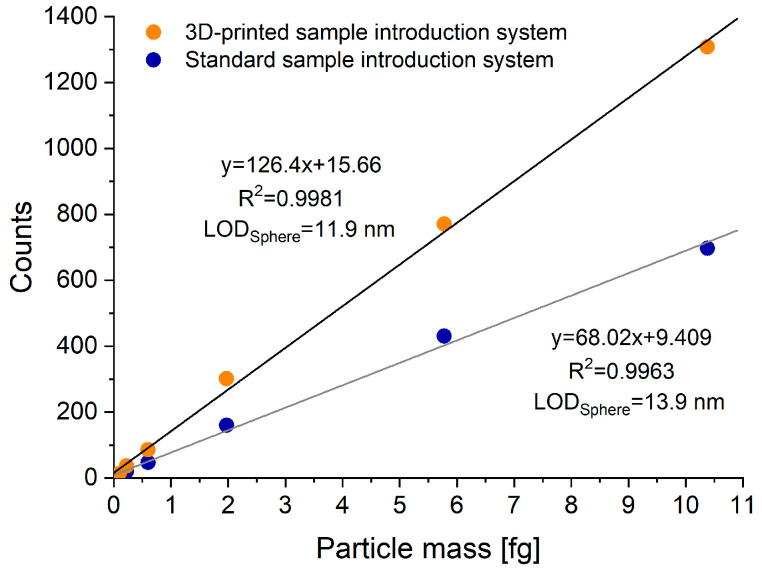
Calibration curves, obtained by using the 3D-printed and the original sample introduction system with the help of a series of Au standard nanoparticles (20 to 80 nm in diameter).

We also conducted a series of experiments with the aim of comparing the signal-to-noise ratios (Figure 9) and the limit of detection (Figure 10) values that could be achieved with the different sample introduction systems. Since the ICP-MS settings were kept the same, differences in any of these metrics come from two characteristics of the compared systems. On one hand, they have substantially different gas and liquid throughput that affect the ionization efficiency of the plasma. Generally, the less energy intensive it is to break down the gross mass flow going through the plasma, the higher is the ionization rate (higher sensitivity), but it also applies to the ionic background signal, therefore the signal-to-noise ratio is virtually unaffected. On the other hand, there is a difference in the net liquid transport into the plasma. Despite the higher particle detection efficiency of the 3D-printed SIS, due to its rather low sample uptake rate, only around 4.1 μL·min^−1^ liquid transport rate is realized via aerosol into the ion source, whereas for the original system this value is around 25.7 μL·min^−1^. The lower this liquid transport rate, the lower is the average background signal (maximum position of the background peak), thus, the signal-to-noise ratio is enhanced. Both effects are observable in Figure 9, since using the new system, the average counts per detection event are found to be higher, while background signals are lower, compared to those values of the original sample introduction system.

Figure 10 illustrates the extent the sensitivity of the instrument and the detection limits of particles can be enhanced by replacing the original sample introduction system with the new one, which is better suited for single particle detection purposes. Using the two calibration curves, the absolute LOD_size_ [35] for pure, zero porosity, spherical Au nanoparticles were found to be 11.9 nm when using the new SIS, and 13.9 nm when using the original one. This, roughly 20% decrease is small, but it can be important in some applications (expressed in particle mass, the improvement is more than 63%).

### 3.4. Applications in Single Cell ICP-MS Analysis

#### 3.4.1. Monitoring the Effects of Nickel Ions on the Proliferation of Chlorella Algal Cells

In this experiment, the suppressing effects of increasing Ni^2+^ concentrations on cell population growth and the cellular accumulation of the Ni^2+^ ion were monitored in Chlorella algal cells by single cell ICP-MS measurements. Four parallel cell cultures were made from the same Chlorella algae strain in different Ni^2+^ containing media, ranging from 0 to 150 μM. All cultures were kept under the same conditions for 72 h, throughout which small samples were taken out from them every 24 h to monitor the actual cell population and Ni^2+^ content of algal cells. The samples were analyzed by spICP-MS measurements, utilizing the new SIS. For cell counting, algal cells were detected at mass 24, relying on their Mg content due to their high chlorophyll levels [36], whereas their Ni content was monitored at mass 58.

**Figure 11:**
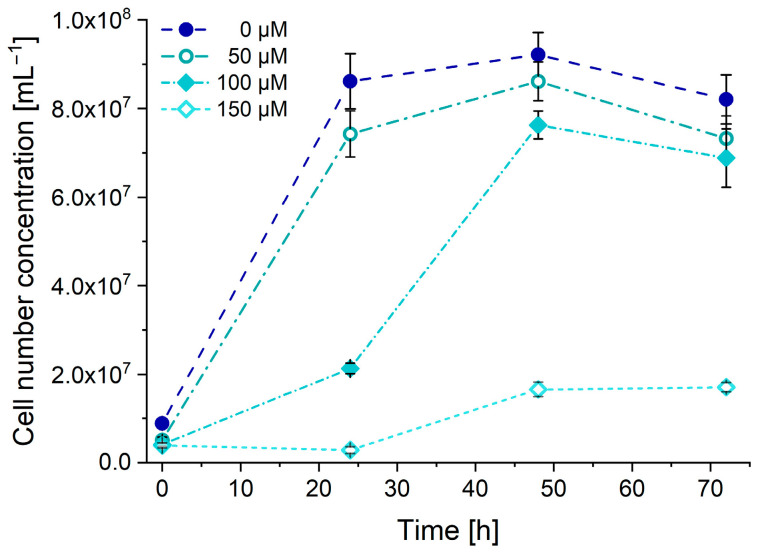
Population of the algae cultures, cultivated with different Ni^2+^ concentrations.

As Figure 11 shows, nickel ions imposed a gradual cell growth suppression effect over the applied concentration range. The culture with the 50 µM of Ni^2+^ is hardly affected, while the one with 100 µM had its population peak delayed by almost a day, whereas a 150 µM Ni^2+^ concentration suppressed the cell growth significantly throughout the whole duration of the experiment. Results showed the nickel content associated with individual cells remained below the detection limit for all samples except for the sample, which was cultivated in the medium, containing the highest Ni^2+^ concentration. After 24 h of cultivation, the average accumulated nickel content of the cells was slightly above 5.0 fg, which gradually increased up to 6.3 fg in the course of the experiment. Determining the nickel content of the individual cells was challenging, since the consumed amounts were just above the limit of detection, which was increasing along with the nickel content absorbed due to higher ionic backgrounds. Considering the tendencies discussed in the previous chapter, determining cellular nickel content would not have been feasible without utilizing the 3D-printed sample introduction system.

#### 3.4.2. Counting of Human Endothelial Cells

In this experiment, the counting of human endothelial cells was realized using the 3D-printed sample introduction system. Endothelial cells after trypsinization were treated with paraformaldehyde to ensure a spherical shape and enhance their durability, both of which facilitated reliable single particle detection. Prior the measurements, the average diameter of the cells was determined via optical microscopy and was found to be 15.4 µm.

A suspension of 1.1·10^6^ endothelial cells per mL was prepared and introduced into the ICP-MS via the new SIS. Individual cell detection was attempted on several different mass numbers associated with crucial and frequent trace elements of cells [37,38], but only ^58^Ni and ^64^Zn were found to be reliable for this purpose. However, using Zn was found to be favorable over Ni in terms of signal-to-noise ratio since this value was nearly twice as good for Zn. As a demonstration of the capability of the single cell ICP-MS technique for the monitoring of the cell count, the original suspension was diluted 3-, 5- and 12-fold and the cell number concentration of these samples were determined by Zn signal-based spICP-MS measurements. Found concentrations corresponded to dilution factors of 3.1, 4.9 and 12.3, respectively, thereby evidencing the practicality of the approach.

It has to be noted though the particle detection efficiency was found to be only 0.65% for these cells. This particle detection efficiency was relatively low compared to the value obtained for nanoparticles (see Section 3.3.2.), but it was expected due to the low transmission of droplets with diameters above 15 µm. However, please note the original sample introduction system had virtually zero transmission in that region, thus it was unable to introduce particles in this size regime (Figure 8).

## 4. Conclusions

The developed completely 3D-printed sample introduction system was found to outperform the standard system in single particle/single cell analysis. It was able to provide enhanced signal-to-noise ratios and particle detection efficiencies due to its ability to enhance sensitivity while suppressing the ionic background. Also, it was able to extend the useful range of particle size that could be analyzed. Although sample introduction systems with higher overall performance have already been reported the literature, our study shows for the first time in the literature that 3D-printing is able to produce complete sample introduction systems, which can have a performance comparable to or even better than the standard ones. It is also clear, however, that some challenges are still associated with the direct production of microfluidic devices by 3D-printing from plastic materials. For example, the printing quality of the finest details of pneumatic nebulizers is still limited by the resolution and support structures of the technology employed. In these applications, the resin is also expected to be trace analytically pure (e.g., free from metal contaminations) and resistant mechanically, as well as chemically. These concurrent requirements are somewhat specific to sample introduction devices meant to be used in ICP-MS analysis, and together are rarely addressed by the developers of 3D printing technology. Thus, further improvements in 3D-printing technology need to be pursued.

## Data Availability

Data can be available on request.
